# Survival and Evolution of CRISPR–Cas System in Prokaryotes and Its Applications

**DOI:** 10.3389/fimmu.2016.00375

**Published:** 2016-09-26

**Authors:** Muhammad Abu Bakr Shabbir, Haihong Hao, Muhammad Zubair Shabbir, Hafiz Iftikhar Hussain, Zahid Iqbal, Saeed Ahmed, Adeel Sattar, Mujahid Iqbal, Jun Li, Zonghui Yuan

**Affiliations:** ^1^MOA Laboratory for Risk Assessment of Quality and Safety of Livestock and Poultry Products, Huazhong Agricultural University, Wuhan, China; ^2^Quality Operations Laboratory at University of Veterinary and Animal SciencesLahore, Pakistan; ^3^National Reference Laboratory of Veterinary Drug Residues (HZAU), Huazhong Agricultural University, Wuhan, China; ^4^MAO Key Laboratory for Detection of Veterinary Drug Residues, Huazhong Agricultural University, Wuhan, China

**Keywords:** CRISPR–Cas, Cas9, sgRNA, gene expression, gene editing

## Abstract

Prokaryotes have developed numerous innate immune mechanisms in order to fend off bacteriophage or plasmid attack. One of these immune systems is clustered regularly interspaced short palindromic repeats (CRISPR). CRISPR-associated proteins play a key role in survival of prokaryotes against invaders, as these systems cleave DNA of foreign genetic elements. Beyond providing immunity, these systems have significant impact in altering the bacterial physiology in term of its virulence and pathogenicity, as well as evolution. Also, due to their diverse nature of functionality, cas9 endoribonuclease can be easily reprogrammed with the help of guide RNAs, showing unprecedented potential and significance for gene editing in treating genetic diseases. Here, we also discuss the use of NgAgo–gDNA system in genome editing of human cells.

## Introduction

In the late 1800s, scientists reported that, in India, water from two rivers, Ganges and Yamuna, have an antibacterial agent that killed *Vibrio cholera* (1). These antibacterial agents, later termed as bacteriophages (from “bacteria” and the Greek word *phagein*, “to devour”), have important role in treatment of various diseases. However, phages still have to attain their therapeutic potential in clinical settings; the role and importance of bacteriophages in medical and environmental science is currently reaching a new crescendo. Marine virologist reported, in 1980s, that 1 L of sea water contains round about 10 billion bacteriophages. Hence, today these viruses (bacteriophages) are generally considered as the most abundantly found diverse biological entities on Earth (2, 3).

The ability of prokaryotes to withstand viral attacks is a key for their survival. With the passage of time, bacteria have managed themselves according to various inhospitable habitats, such as harsh environmental conditions and various bacteriophages attack. Bacteria are able to withstand these challenging conditions due to their flexibility in their genetic repertoire and genome homeostasis and avert bacteriophage predation with the help of their defense system. One important point regarding eubacteria is that they have managed to control their genome size by balancing the uptake of material that is beneficial along with tactical loss of redundant genes, in a dynamically designed dance with offensive mobile genetic elements (MGEs) like plasmids, viruses, and transposons (4). However unlike eukaryotes, prokaryotes have the ability to orchestrate this without expansion of their genome. Recently, it was discovered that clustered regularly interspaced short palindromic repeats (CRISPR) and its related proteins (cas) play vital role in providing adaptive immunity in prokaryotes and archaea against viruses and plasmids, and it has been established that this system serves as a guardian of bacterial genomes (5, 6).

This system has been found within 90% of archaeal and 45% of bacterial genomes that play role as an immunity-like resistance in these microorganisms against viruses and plasmids (7). The CRISPR–cas system consists of a genetic locus that contains the CRISPRs, non-repetitive, unique spacer sequences, and adjacent 6–20 genes encoding the cas (CRISPR-associated) proteins (8). The repeats that are present within each CRISPR locus are highly conserved and their base pairs range varies between 23 and 47. However, in case of spacers, the range of base pairs varies between 21 and 72, and these are composed of extrachromosomal elements (9). In most cases, the CRISPR loci contains less than 50 repeat/spacer units, but, in some species, like *Chloroflexus* spp., contain repeat/spacer units up to 375 (9). CRISPR–cas locus can also be located in a microbial genome. For example, the genome of *Methanocaldococcus jannaschii* contains 18 CRISPR–cas loci (8).

However, recently conducted studies revealed that CRISPR system has other functional roles beyond adaptive immunity. Due to novel roles of CRISPR systems in which these systems not only expand functional repertoire but also point out the need to manage safe-keeping of genome integrity and uptake of MGEs that are beneficial for adaptive purposes. Here, in this review, we not only discuss CRISPR–cas system role in adaptive immunity of prokaryotes along with their role in altering the bacterial physiology but also discuss CRISPR systems impact in the evolution of bacterial genome, regulation of gene expression, as well as their use in various medical and bio-engineering fields.

## History of CRISPR–Cas System

Some researchers in 1987 cloned and sequenced the *iap* gene that is present in *Escherichia coli* and responsible for the conversion of alkaline phosphatase isoenzyme (10). Later, a subsequent study was conducted on the iap gene by scientists, and they reported that set of 29-nucleotide (nt) repeats separated by unrelated, non-repetitive, short sequences called as spacers (11). So, this was the first report about CRISPR locus and, later, same findings has been also observed, when scientists did gene or complete genome sequencing in bacteria and archaea (12–15). This availability of knowledge about genomic sequences helps in the identification of CRISPRs that are present in many such species (16).

For this particular structure of loci, the term CRISPR was used first time in 2002 (17). Typically, a repeat cluster has an AT-rich region, which is present at leader sequence; in intra-species, this sequence is 100 base pair long, but not in interspecies (17). A set of protein coding genes also known as CRISPR-associated (cas) genes are normally present on one side of loci. Spacer sequence analysis in various CRISPR loci showed that these spacers have identical sequences to invader’s genetic elements such as plasmids and bacteriophages (18, 19). In the early studies, it was revealed that there is a relationship among phage sensitivity and the absence of spacers, matching the sequence against that specific phage, indicating the role of CRISPR loci in immune function (18). The CRISPR loci on comparison from various *Yersinia pestis* strains indicates that spacer acquisition occur in a polarized fashion in term of new units and are added at leader end of cluster (19). In the light of these studies, it was revealed that there is an existence of mechanism which not only exploits the potential of nucleic acid base-pairing but also enables the sequence-based interfering of gene expression, phage infection, or both. With the help of detailed bioinformatics analysis of cas genes, this possibility was supported, and it showed a bias toward proteins that are anticipated to facilitate nucleic acids transactions, led to the suggestion that CRISPR immunity might work in a similar way as the eukaryotic RNAi, that also uses nucleic acid sequences in order to control gene-silencing pathway (4, 8).

In 2007, a study was conducted on phage infection in *Streptococcus thermophiles*, which revealed that CRISPR loci have key role in adaptive immunity (20). This study gives the first experimental proof that sequence similarity among spacer and protospacer is a key for CRISPR immunity. More recently, it was revealed that CRISPR-based interference can also limit plasmid conjugation in *Staphylococcus epidermidis*, which indicates wide spectrum role of CRISPR system in the prevention of horizontal gene transfer (HGT) in bacteria (21).

## Fate of CRISPR System Against Invading DNA

**Figure 1:**
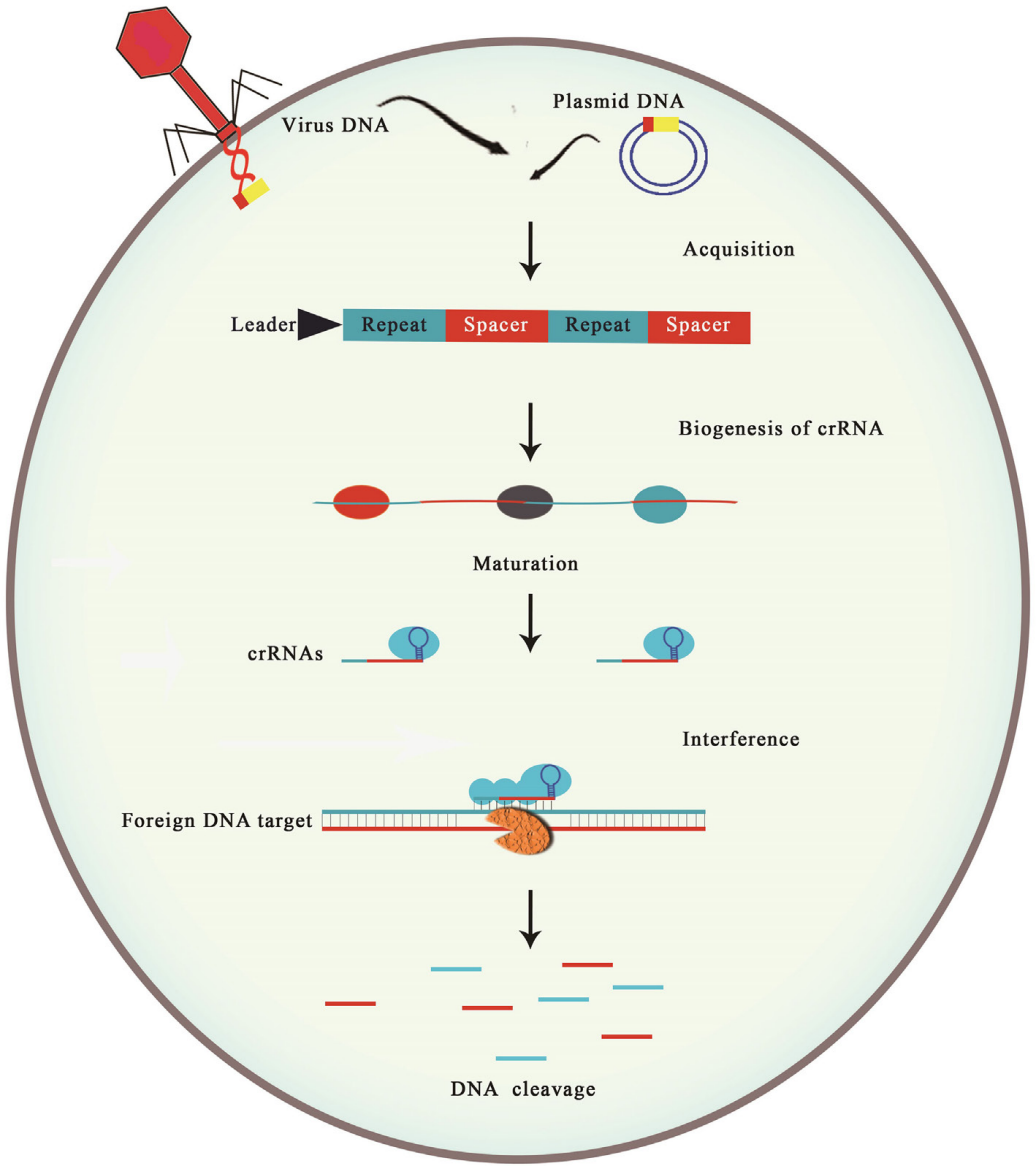
**Mechanism of CRISPR–cas immunity divided into three stages**. Stage 1: spacer acquisition. In the first stage, specific fragments of virus or plasmid double stranded are integrated at the leader end of CRISPR array on host DNA. A CRISPR array consists of unique spacer (red box) interspaced between repeats (blue box). Spacer acquisition occurs in the presence of cas1 and cas2 proteins, which are present near the vicinity of CRISPR array. Stage 2: biogenesis of crRNA. In this stage, RNA polymerase at leader end helps in the transcription of Pre-CRISPR RNA (Pre-crRNA) to mature crRNA. Stage 3: interference. In the final stage, specific match between crRNA spacer and target sequence leads to the cleavage of foreign genetic elements (blue and red strips).

The mechanism of CRISPR–cas system can be categorized into three stages as shown in Figure 1. First step: which is also called as adaptation (22, 23), immunization (9), or acquisition of spacer (24, 25), involves the spacer integration between two contiguous repeat units that are present within the CRISPR locus after the recognition. Primarily, the spacers are incorporated at leader end of CRISPR locus; therefore, position of spacer in locus gives the information about its acquisition event. Incorporation of viral genome sequence (Spacer) into CRISPR locus termed as protospacer (26). In most cases, a very short section of conserved nucleotides present near the protospacer vicinity, called as CRISPR motif or protospacer adjacent motif (PAM), it appears that this motif is very essential in the process of DNA fragment acquisition (27, 28). Cas1 and cas2 are the key proteins required in first phase because these proteins have significant role in spacer acquisition in those genomes which have CRISPR–cas system.

Clustered regularly interspaced short palindromic repeats expression is the second stage of this system immune mechanism. In this stage, transcription of pre-CRISPR RNA (pre-crRNA) occurs with the help of RNA polymerase (RNAP) from the CRISPR locus. After the transcription, pre-crRNA cleaves with the help of specific endoribonucleases into small CRISPR RNAs (crRNAs). On the basis of their function, these crRNAs are termed as guide RNAs (29, 30) or prokaryotic silencing RNA (psiRNAs) (4, 31). In the final stage, which is also referred as interference (27) or immunity (22), multi-protein complex having mature crRNAs can recognize and form base pair, which is specific to incoming invader’s DNA or RNA having perfect or almost perfect complementarity (29). This helps in initiating the cleavage of crRNA–invader nucleic acid complex (22). However, if there are any mismatches between target DNA and spacer or there is any mutation in the PAM, then the process of cleavage is not initiated. In this circumstance, viral replication proceeds as targeting of DNA is not possible, leading to susceptibility of host to the virus attack, resulting in lysis of host. As a result of this, viruses released from lysed host cells can kill other susceptible host cells. In order to properly operate as a defense system, functionality of all three stages is a key; however, it is also important to note that each stage is not dependent on the other stage, both temporally and mechanistically.

## Classification of CRISPR-Cas System

Cas1 and cas2 proteins are genetically present throughout all types and subtypes, but there are also some proteins that are present specifically in each system. Like, cas3 protein present in type I, cas9 protein present in type II, and cas10 protein present only in type III system. Up till now, phylogenetically, only type II systems have been identified in bacteria, but there is a bias about type I systems and type III systems in bacteria, hyperthermophiles, and archaea, respectively.

## Type I CRISPR System

Most widely distributed system in archaea and bacteria is CRISPR–cas system type I (32). This system consists of six subtypes (A–F), all of these subtypes have cas3 gene. This gene has two domain: N-terminal HD phosphohydrolase domain and second one is a C-terminal DExH helicase domain (4, 32). However, in some subtypes like A, B, and D, there are other genes that encode both nuclease and helicase domain. In all subtypes of this system, these two domain work together by cleaving the HD domain and unwinding of helicase domain in order to degrade DNA. However, as cas3 alone unable to recognize the invading DNA, so it also cannot defend cells against infection (29, 33). In each subtypes of this system, there are specific subtype cas proteins that assemble into crRNA-guided surveillance complexes. These complexes help in finding and binding of the target sequences which are complementary to the crRNA spacer.

**Figure 2:**
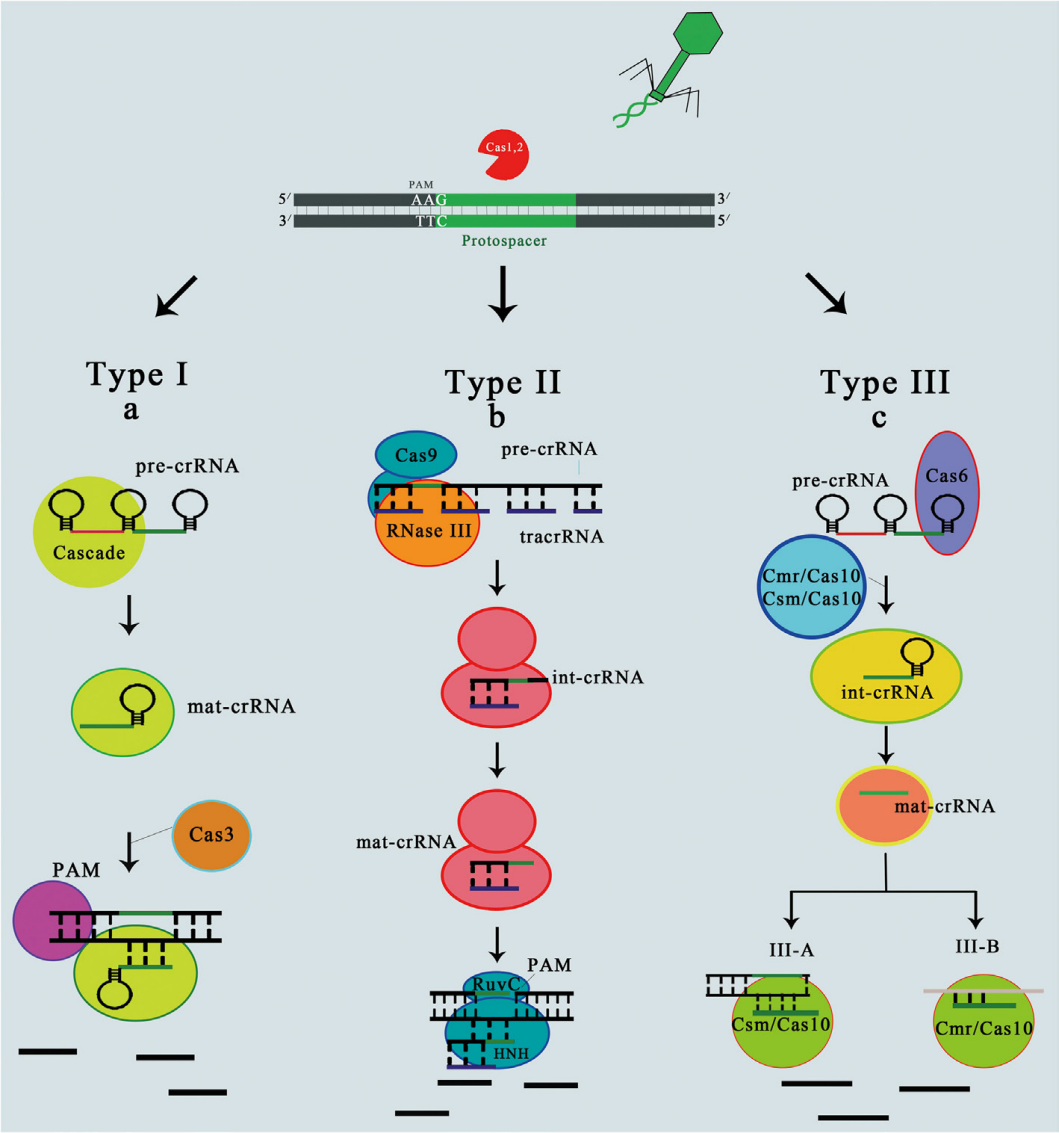
**Mechanism of CRISPR immunity in all three types of CRISPR–cas system**. In type I system **(A)**, after the spacer acquisition in the presence of Cas1 and cas2 protein, biogenesis or processing of Pre-crRNA within the Cascade complex is achieved by cas6 endoribonuclease. Cleavage occurs at the base of stem-loop formed by repeat RNA to release mat-crRNA. After that cascade having mat-crRNA recruits the cas3 nuclease in order to nick the DNA strand complement to the protospacer, immediately downstream of the interaction region with crRNA spacer. This leads to the breakdown of foreign DNA (black strips). In type II system **(B)**, after spacer acquisition, this system utilizes tracrRNA for the biogenesis of crRNA. Pairing between tracrRNA and Pre-crRNA results in the formation of double strand substrate, which is cleaved by the host encoded RNase III in the presence of cas9 to liberate the small crRNA (int-crRNA). After the liberation of int-crRNA, there is immediate trimming of int-crRNA at 5′ end that yield to mat-crRNA. For target cleavage, crRNA, tracrRNA, and cas9 domains (Ruvc and HNH) are necessary. HNH domain helps in cleaving the DNA strand that is complementary to crRNA guide, while Ruvc domain responsible for cleaving the non-complementary strand. In type III system **(C)**, processing of Pre-crRNA to int-crRNA occur in the presence of cas6. After that int-crRNAs are incorporated into Csm/cas10 or Cmr/cas10 complex, where further maturation occur by trimming at 3′ end sequences that results in the liberation of mat-crRNA. Genetic evidence revealed that DNA cleavage occur by sub-type III-A (37), while on the basis of biochemical data, it was revealed that subtype III-B helps in cleaving the RNA molecules (31).

The first crRNA-guided surveillance complex for antiviral defense was described in *E. coli* K12 (type I-E) (29, 33). This complex is made up of five functionally important Cas proteins (33). Cas6e is an endoribonuclease (previously known as CasE or Cse3) that helps in cleaving the long CRISPR RNA into mature 61-nt crRNAs (29, 34). This mature crRNA remains attached with the CASCADE complex, helps in detection of DNA match, and cleaves the foreign DNA when found (35) (Figure 2A). This crRNA and Cas6e are necessary for the stability and assembly of Cse1, Cse2, Cas7, and Cas5. Some researchers recently determined Cascade complex structure with the help of cryo-electron microscopy (cryo-EM) (36). So, the detailed study of this structure helps us in understanding how this crRNA is protected by the cas proteins and also makes possible for crRNA to form base pair which is complementary to the foreign nucleic acid. As crRNA forms a complementary base pair with target DNA strand, relocating non-complementary strand results in the formation of R loop like structure (33).

This crRNA-guided surveillance complexes have been found in various subtypes of type I system, such as in *S. solfataricus* (type I-A) (38), *Bacillus halodurans* (type I-C) (39), and *Pseudomonas aeruginosa* (type I-F) (36).

## Type II CRISPR System

This system has four genes: cas1, cas2, cas9, and csn2 in case of type IIA or *cas4* in case of type II-B. However, the cas9 is a signature gene of this system that encodes a multifunctional protein that not only play role in the biogenesis of crRNA but also cleaves foreign DNA (40, 41). The biogenesis of crRNA in type II system is different as compared with other two systems, because trans-activating crRNA (tracrRNA) is necessary for this system. In *Streptococcus pyogenes*, tracrRNA is encoded upstreamly and found on reverse strand of the CRISPR–cas locus (40). Fusion of tracrRNA and crRNA leads to the formation of dsRNA that is responsible for cleaving the RNase III enzyme in cell. Although biogenesis of crRNA stops upon cas9 deletion, its specific role in this process is not clear (40). However, it was recently revealed that for the denaturation of target DNA, cas9 require mature crRNA and the tracrRNA (41). Cas9 proteins consists of HNH and RuvC domain, former domain helps in cleaving the DNA strand which is complementary to the guide crRNA; however, latter domain helps in cleaving the strand that is non-complementary (41) as shown in Figure 2B.

## Type III CRISPR System

Type III system have two subtypes; type III-A and type III-B (32). These systems are most commonly found in archaea. Both subtypes of this system have cas10 and cas6 genes. Cas6 also known as endoribonuclease, which is CRISPR-specific, and cas10 play role in target interference. Although two subtypes have these similarities, still it appears that these two systems target chemically different substrates, like *S. epidermidis* have type III-A system and target of this system is DNA (21) and *Pyrococcus furiosus* and *S. solfataricus* have type III-B systems that target RNA (31, 42, 43) (Figure 2C). This basic variation about target indicate us functional diversity, which is present within the same CRISPR–cas type.

## Role of CRISPR Beyond Adaptive Immunity of Prokaryotes

Latest studies about CRISPR system, opens a new gateway, which gives us the information that this system has role beyond adaptive immunity of prokaryotes (44). There is explosion of reports about CRISPR–cas system that helps in the analysis of cost and benefits of this system and also expands our knowledge in understanding the various roles they play in bacteria. A new perception about functions of CRISPR system has evolved, as it has the ability to not only control endogenously transcription but also regulates bacterial pathogenicity. A recent study gives us information that this system has the ability to regulate endogenous genes, responsible for pathogenesis and virulence of *Francisella novicida* (45, 46). It was also shown that type II CRISPR system can repress bacterial lipoprotein transcription that leads to the pro-inflammatory response in human host. Incongruously, this immune system in bacteria play role in the regulation of those genes, which are responsible for encoding of factors that have impact in pathogenesis of bacteria. CRISPR immune system also helps bacterium to bypass the human immune system. Similarly, very recently, it was revealed that CRISPR can change the pathogenic behavior of *Campylobacter jejuni* (47). In *Neisseria meningitidis* pathogenesis and *Legionella pneumophila* infection also same outcomes has been observed by this system (48, 49) (Table 1).

**Table 1:** **Bacterial species and their pathogenesis with experimental model**.

Bacterial species	Infection	Experimental model	Reference
*Campylobacter jejuni*	Guillain Barre syndrome	Human intestinal epithelial and HT-29 cells	(47)
*Legionella pneumophila*	Legionnaires’ disease	Macrophages and aquatic amoebae	(49)
*Neisseria meningitidis*	Meningococcal disease	ND	(48)
*Francisella novicida*	Tularemia	Mice	(46)

*ND, not described*.

## The Evolution of CRISPR System Playing as a Defense

Several studies on role of CRISPR system working as defense system in bacteria and archaea gives us a clue that how this system in general interplay coevolutionary between prokaryotes and their bacteriophages, and specifically speaking provide information about their genetic arms race (50–53). Also, different studies showed that, upon the exposure of viruses, these CRISPR loci diversify rapidly in response to that particular exposure or virus attack (54, 55). In turn, bacteriophages also mutate in order to bypass the CRISPR-based immunity (26, 51). In the light of recent study, which has shown a twisted turn of event that phage not only acquire but also use this CRISPR system of bacterium in their host in order to target antiviral defense system, illuminating the evolutionary worth of this system (56). Hence, these studies indicate that high frequency rate of mutation were observed in viral genomes, host-virus population ecology, with indications that CRISPR immune systems are a key force for the sustainability of bacterial and evolution of viral genome as well.

Clustered regularly interspaced short palindromic repeats system protects chromosome against invasive genetic element in order to maintain genetic homeostasis, which, in turn, is beneficial for the cell, because this system act as a barrier against acquisition of foreign element (57, 58). No doubt these invasive elements, such as plasmids, phage, and other conjugative elements, sometimes carry beneficial genes, which not only play positive role in bacterial adaptation but also have an evolutionary benefit that helps in increasing its fitness in the environment, like antibiotic resistance and virulence factors. A recent study reported that targeting of CRISPR sequence in bacterial chromosome results in the loss of pathogenicity islands (59). This spread is persistent not only in case of plasmid encoded-antibiotic resistance cassettes but also found in case of HGT of pathogenicity islands among different bacterial genomes. There are many examples that indicates establishment of negative correlation between the occurrence as well as diversity of CRISPR system, and existence of phages and plasmids, as illustrated in *Campylobacter, Enterococcus* and many group A *Streptococcus* species (60). However, occurrence of CRISPR loci in *Enterococcus faecalis* and *Enterococcus faecium* genome is rare, and typically 25% of their genome constitute of MGEs (60, 61). This can be correlated with the ability of CRISPR–cas systems in which this system interfere directly with natural transformation, as shown in *Staphylococcus* (21, 37). More precisely speaking, targeting of conjugative plasmids by CRISPR system leads to the adverse effect on antibiotic resistance in *S. epidermidis* (37, 57). On the other hand, the paucity of CRISPR loci in *Staphylococcus aureus* likely correlates with plasmids occurrence and other MGEs that seems to increase the virulence in this pathogen (57, 60). Almost same findings have been observed in *pneumococci*, as experiments revealed that CRISPR system can avert the switching of capsule for successful infection by *Streptococcus pneumoniae* (59). In terms of cost, when CRISPR system plays as a defense in the bacterium, it can result in the loss of key phenotype, such as virulence in human pathogens. This trade-off might be a possible reason as to why this system existed “only” in half of the bacterial population. Moreover, the existence and activity of CRISPR immune system indicates not only frequency but also richness of ecosystem with predators and invaders, the intensity of CRISPR-mediated arms race between virus and host, as well as the existence of other defense systems in host, such as restriction–modification, surface receptor mutation, and abortive infection.

## CRISPR System and Its Regulation

The key function of CRISPR system is to provide immunity in prokaryotes, and the expression of this system’s defense mechanism might be due to invasion of extra chromosomal elements. In a recent study, it was proven by the approach of shotgun proteomic study in *S. thermophiles* that, after phage infection, there is an increase in the expression of cas protein (62). Similarly, in *Thermus thermophiles*, there is an increase in CRISPR expression after phage infection (63). Moreover, in response to UV, CRISPR system can be modified which play role in DNA damage sensitivity, pointing out its other possible roles in addition to neutralization of foreign genetic elements (64, 65). However, up to date, very little information is available about CRISPR system regulation in response to foreign stimuli and also the periods during which this system is not required (64, 65). In case of availability of beneficial elements, it is favorable to downregulate the CRISPR system. However, upon phage exposure, most evidence supports CRISPR–cas upregulation. As we all know, effectiveness of CRISPR system is not 100%, because some beneficial elements will need to be acquiring in order to maintain their selective advantage.

Most information about regulation of CRISPR system is available for *Salmonella enterica* serovar Typhi and *E. coli* having type I-E systems. It is established universally that Histone like nucleotide structuring protein (H-NS) is a regulator, which plays role in bacterial chromosome compacting with the help of an AT-rich, curved DNA (66). Several promoters are located in the close vicinity of curved DNA. Binding of these promoters with H-NS leads to the inhibition of RNAP binding, resulting in gene-silencing (66). In *E. coli*, binding sites of H-NS is present near cas operon promotor, so cas gene expression are negatively regulated by H-NS (67, 68). It is hypothesized that, when foreign genetic elements invade their nucleic acid in the cell, binding between H-NS and invading nucleic acid will occur at that time, which is due to higher AT-content (69, 70). It is predicted that Sequestration of H-NS results in the release of cas and/or LeuO promoter which are recognized by RNAP and that leads to activation of expression, allowing active CRISPR–cas-mediated defense (67, 68, 71).

In *S. typhi*, negative regulator for cas expression is lysine-responsive regulatory protein (LRP) (72). This protein also works like H-NS as LRP binds with cas promoter that results in the inhibition of RNAP binding with cas promoter. However, LRP has the ability to bind along with H-NS, indicating that these two proteins can interact together which results in generation of nucleosome structure that leads to the of cas gene (72). It is important to note that LRP has no role in regulating the cas operon in *E. coli*, indicating that type I-E CRISPR–cas systems work differently at regulatory level in these two closely related organisms (67, 72).

LeuO, a LysR-type transcriptional regulator, also competes with H-NS in *E. coli* and *S. Typhi*, in response to amino acid starvation that also results in the repression of cas gene (68, 72, 73). LeuO usually binds at flanking region of the cas promoter and as well as H-NS binding site. LeuO competes with H-NS for binding with DNA, enabling promoter to recognize by RNAP, which facilitates cas gene expression (68, 72, 73). During the state of amino acid starvation, LeuO expression is increased due to aggregation of small molecules of guanosine 3′-diphosphate 5′-triphosphate and guanosine 3′,5′-bis (diphosphate) also known as (p) ppGpp (74). However, it is interesting to note that when *E. coli* get infected with phage lambda, then there is no accumulation of (p) ppGpp (75). In theory, there is a possibility that amino acid starvation can be triggered by other phages infection, which results in the LeuO-dependent CRISPR–cas system activation.

**Figure 3:**
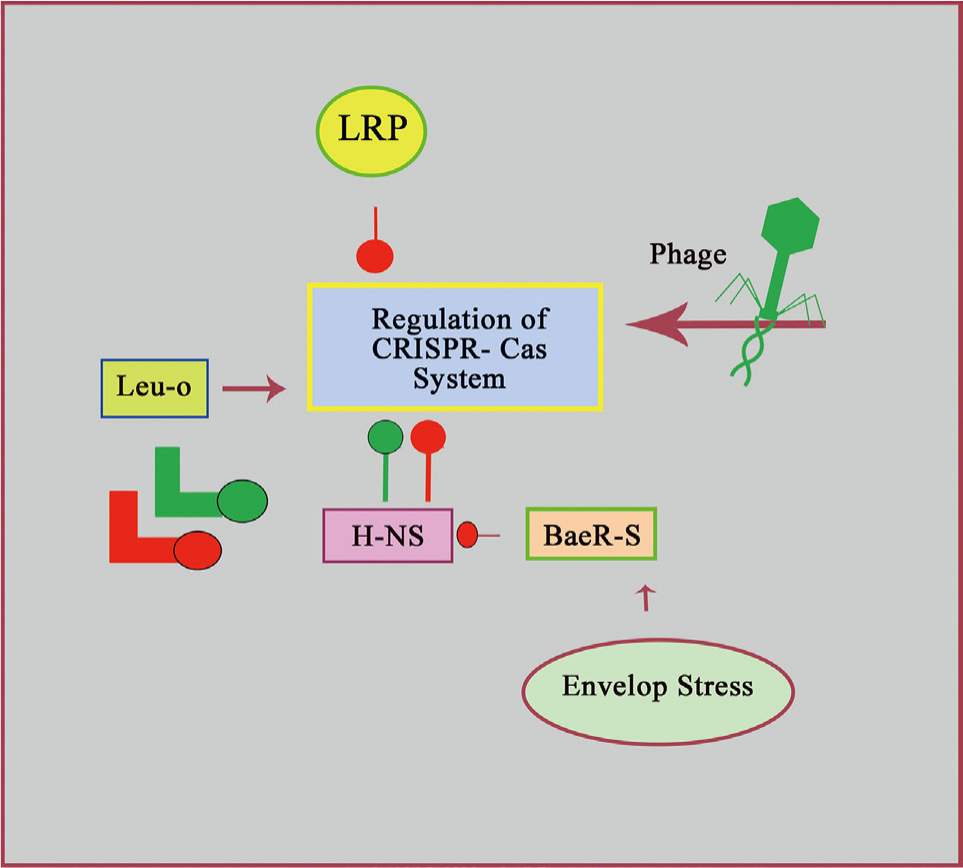
**Regulation of CRISPR–cas system**. This network model indicates the summary of CRISPR–cas system regulation. Green (*E. coli*) and red (*Salmonella*) lines with round head indicate negative effects on CRISPR–cas system regulation. While, triangular arrow head indicates positive effect.

During the envelop stress, two-component regulatory system (BaeR-S) becomes activated (76). Phage infection causes viral protein accumulation in membrane that leads to the envelop stress. In response to membrane stress, activation of histidine sensor kinase BaeS is triggered by phosphorylation, which leads to the activation of BaeR protein. After the activation of BaeR, this protein enable DNA binding, modulating gene expression (76). In *E. coli*, binding site of BaeR is located near the casA promoter of H-NS binding site. Therefore, binding of BaeR act as an antagonist for H-NS binding that leads to release of cas promoters for the recognition of RNAP and cas expression (77, 78) as shown in Figure 3.

In summary, regulation of CRISPR–cas systems occurs at transcriptional level of cas genes and CRISPR arrays at post-transcriptional level on cas proteins. Hence, picture of these systems, how they are regulated is diverse and far from complete.

## Gene Regulation by CRISPR and Its Role in Pathogenesis

*Francisella novicida* is an intracellular bacterial pathogen that causes a relatively rare human disease by bypassing the innate immune system of host (79). *F. novicida* has various mechanisms, and, with help of these mechanisms, this bacterium subverts the host macrophages as well as other immune cells functions. After the engulfment of *F. novicida* by macrophage, this bacterium enters phagosome, a compartment having several antimicrobials and innate immune recognition receptors (79). Toll-like Receptor 2 (TLR2) is an example of innate immune receptor, which detects BLPs (80, 81). Activation of TLR2 leads to a pro-inflammatory response that recruits and activates immune cells, which results in combating and clearing of the bacterial pathogen.

The expression of BLP is repressed in *F. novicida*, because this pathogen uses cas9, tracrRNA, and scaRNA as regulator which ultimately leads to lowering of BLP level by roughly 2-fold in its envelop (45, 82). As a result of this, activation of TLR2 is dampened, and it facilitates survivability of pathogen within the host. If regulation is not mediated by CRISPR system, *F. novicida* elicits an inflammatory response that is TLR2-dependent, because deletion mutants of cas9, tracrRNA, and sacRNA induce more inflammatory response as compared with wild-type bacteria (45). This inflammatory response is dependent on both TLR2 and overexpression of BLP, as strains lacking the regulatory components so BLP initiates a response that is limited only to wild-type levels (45, 82). Moreover, deletion mutants are highly attenuated (over 1000 fold) as they lack the CRISPR–cas system (45). Additionally, cas9, tracrRNA, and scaRNA deletion mutants are not able to cause lethal infection in mice, which again emphasizes their importance as virulence regulators in *F. novicida* (45).

Currently, in *F. novicida*, cas9 is the only known example of CRISPR–cas system that acts naturally in a regulatory capacity; there are also other observations about other species that use cas9 as a virulence factor. In a human lung epithelial cell model, in order to attach the *Neisseria meningitidis* to cell surface of the host, cas9 is the key for not only invasion but also for intracellular replication (45). Also, in a colorectal epithelial cell model, cas9 is the key for the attachment as well as invasion of *C. jejuni* (47). The exact mechanisms by which cas9 involved as a virulence factor in these organisms is yet to be known. However, on the basis of cas9-established role as a regulator of gene expression in *F. novicida*, it seems that cas9 work in combination with tracrRNA or an unidentified small RNA, and regulate specific transcription, which eventually play role in the control of virulence properties.

Moreover, cas9 role is correlated with the virulence of *Campylobacter*-specific strains, which encodes the Cst-II sialyl transferase, and this Cst-II produces a sialylated lipooligosaccharide. However, cas9 deletion in *C. jejuni* isolates from GBS patients results in the loss of ability to translocate across epithelial cells of intestine. Also, cas9 deleted mutants having sialylated LOS that bind to human serum more strongly as compared non-mutants, which indicate its importance in virulence (47). Interestingly, it is hypothesize that in *C. jejuni*, regulation of CRISPR system may not only help to attach efficiently with host cells but also mask the surface in order to avoid detection by host receptors that results in prevention of immune system activation, like the complement system. Since it is known that, in *Francisella*, membrane BLP is a regulatory target of cas9, and these additional examples of cas9 contribution to virulence traits which play role in attachment of bacteria to the cell surface of host, so it is speculated that CRISPR–cas systems might involve in the composition of envelop.

## CRISPR–Cas System Facilitates Evolution of the Genome

In addition to the regulation of gene expression, CRISPR system can play role in the evolution of genome by self-targeting. However, as CRISPR–cas systems mostly cleave DNA, the chromosomal sequences targeting results in cytotoxicity (59, 83, 84). CRISPR locus can also be targeted during self-targeting of the chromosome (which, by definition, is complementary to the CRISPR transcript), or it can also be targeted when foreign genomic sequences are incorporated in the form of spacer into CRISPR locus, and both these conditions results in the cleavage of genome. Self-targeting that leads to cytotoxicity results in the selection of avoidance mechanisms, which helps in differentiation of self (CRISPR spacer) and non-self (protospacer) (85, 86). Spacers that are derived from chromosome still pose a serious threat to the survivability of cell (59), but bioinformatics and experimental analyses has revealed that self-derived acquisition of spacer events occur repeatedly (84, 87–89). Generally self-targeting events are lethal, but, in some cases, cells can survive as they acquire mutations that led to the inactivation of self-targeting. These mutations can also be found in cas genes, spacers, repeats, and protospacer targets, and there may be involvement of large-scale genome rearrangements, presumably owing to repairing of re-combinational DNA, following genomic cleavage by CRISPR–cas system (59). For example, in *Pectobacterium atrosepticum*, crRNAs target horizontally acquired chromosomal island that is considered as lethal. But, surviving mutants have several chromosomal deletions, as well as complete removal of targeted ~100 kb island that play role in plant pathogenicity (59). Similarly, other chromosomal deletions were also detected when CRISPR–cas target other core genes such as lacZ (59). Self-targeting could actually be beneficial in those rare cases in which rearrangement of the genome confers a fitness benefit.

## CRISPR-Based Applications

Due to the genetic polymorphism nature of cas genes, along with encoding of functionally diverse proteins has set the platform for a wide array of applications.

The CRISPR loci can be used for genotyping of prokaryotes, such as by the exploitation of CRISPR repeat occurrence and their diversity that helps in identification or typing of *Yersinia* and *Mycobacterium* isolates (19), as defined by spacer oligo typing (spoligotyping) (90). Subsequently, spacer content was used for genotypic of which provides insight into the strains common origin as defined by up keeping of ancestral spacers (91). Additionally, polarized spacer at leader end of CRISPR locus act as a genetic tape record for immunization events and it provides basis for tracking the genetic trajectory of a strain. Among others, several bacteria were genotyped with the help of these CRISPR loci, such as *Corynebacterium, Yersinia, Mycobacterium, Pseudomonas, Streptococci, Legionella, Salmonella, Escherichia*, and *Lactobacillus* (92, 93). By using this approach, it is easy to determine the relatedness of pathogenically important strains, such as *Salmonella* and *E. coli* in case of food outbreaks (93).

## Genome Engineering

In the early models of CRISPR systems, it was hypothesized that interference was RNA-mediated and protein-dependent, akin to the eukaryotic RNA interference mechanism (4). However, it was later established that CRISPR–cas primary target is DNA (21), and the interference which cleaves DNA is sequence-specific (21, 22). These findings opened a new gateway for this system subsequent use as programmable nucleases, with many integral biotechnological applications.

Due to the detailed study of CRISPR immunity, particularly type II, cas9-mediated immunity, led to the realization of potential use of this enzyme in genetic engineering (40, 41, 58, 94). In the year 2013, there was explosion of reports revealing the use of cas9 in genome editing, modulation of gene expression, and genetic screening (95), which we discuss briefly below.

## Use of CRISPR–Cas9 as a Tool of Genome Editing in Disease

The CRISPR–cas9 system is an excellent and versatile tool for genomic studies in cells, as this system can be used in dissecting the gene function in various biological processes and diseases as well (96–98). CRISPR–cas9 system makes genome editing as a simple technique, which is before a major technical challenge. Before the use of CRISPR–cas9 system as a genome-editing tool, Zinc finger nucleases (ZFNs) and Transcriptional activator-like effector nucleases (TALENs) were used for genome editing. These two systems use proteins for the recognition of specific sequences in the genomic regions; however, CRISPR–cas system use only sgRNA for editing. The simple and easy use of sgRNA has led to worldwide acceptance for genome editing. The CRISPR–cas system now use as an alternative of ZFNs and TALENs due to its adaptability, simpler in assembly, as well as higher specificity and efficiency (99). Unlike ZFNs and TALENs, CRISPR–cas9 target specificity is determined by the DNA complementary sequence of sgRNA, which helps in easy construction of knockout reagents. For example, this system has been used in creation of knockout of the CCR5 and C4BPB genes in human myeloid leukemia K562 cells (100).

Over the past decade, there are explosion of reports about the studies of human cancer at molecular level; however, there is still need to understand that which mutations play their part in the initiation and progression of tumor. Noteworthy, CRISPR–cas9 system can target complex genetic diseases such as cancer, because this technology has ability to target multiple mutations at the same time. Such as cas9 can be used with combination of sgRNA in order to target more than one genomic loci (101, 102). Recently, some scientists developed CRISPR–cas9-based approach in which they investigate the genes responsible for cancer in mouse model (103).

The CRISPR–cas9 system has been also used for the generation of tumor-associated chromosomal translocations, this occurs during the carcinogenesis *via* illegitimate non-homologous joining of two chromosomes. As CRISPR–cas9 has the ability to cause double strand breaks at precisely defined positions that enables cancer cell lines and primary cells generation with chromosomal translocations and these cells found to replicate in cancers such as Ewing’s sarcoma, AML (104), and lung cancer (105). Recently, another group of scientists developed an efficient *in vivo* method that results in the specific chromosomal rearrangements, when they introduce CRISPR–cas9 system to somatic cells of adult mice (106). In NSCLC (Human non-small cell lung cancers), an oncogene namely EML4-ALK is present, and CRISPR–cas9 system was used to create a mouse model of Eml4-Alk-driven lung cancer. The resulting tumors were inverse of Eml4-Alk, express the Eml4-Alk fusion gene that display histopathological as well as molecular features akin to human NSLCs, and respond with treatment of ALK inhibitors.

This technology has an unresolved issue called as off-target effects. In order to overcome off-target effects, researchers has developed new technology in which they use the mutated active site of RuvC which converts cas9 into a nickase, by using two-paired guides, which direct nicking at two adjacent sites only in a desired target sequence (107–109). In spite of this caveat, cas9-directed genome editing has been successfully used in a wide range of hosts and cell lines that include human cells, plants, rats, mice, zebrafish, nematodes, yeast, bacteria, and also many other organisms (95), creating a revolution in molecular biology.

## Use of CRISPR–Cas9 in Curing Human Genetic Diseases

Gene therapy can be used for curing human genetic disease by correcting the mutations, which play role in causing disease. Recently, there is a ground-break study about CRISPR–cas9 technology, in which scientists repaired cystic fibrosis trans-membrane conductance regulator (CFTR) locus in those patients having cystic fibrosis by homologous recombination in cultured intestinal stem cells. CFTR is a genetic disorder that primarily affects lung and digestive system (110). After correction of CFTR locus by CRISPR–cas9, it was found out that corrected gene not only expressed but also fully functional, as CFTR locus retain its ability of cAMP-induced intestinal stem cell organoid swelling, that is lost in CFTR-mutated cystic fibrosis patients. Hence, this study reveals that there is possibility of curing patients who have monogenic hereditary defect by correction of gene in stem cells. In another study, in which scientists adopt *in vitro* method, they successfully transplanted organoids into the colons of mice (111). These two studies reveal the potential on future of gene therapy in those patients having cystic fibrosis (110).

In human beings, one of the most common genetic diseases is β-thalassemia that is caused by mutation in human hemoglobin beta (HBB) gene. Recently, a study reveals that CRISPR–cas9 cleave HBB gene with combination of piggy-Bac transposon (MGEs that efficiently transposes between vectors and chromosomes), which helps in correcting the mutation of two different β-thalassemia, resulting in conversion of homozygous β-thalassemia to heterozygous in induced pluripotent stem cells (iPSCs) from those patients having this disease (112). During the correction of iPSCs, no off-target effects were detected and cells retain its full pluripotency and also exhibit their normal karyotypes. In culture, when differentiated into erythroblasts, it was revealed that these gene-corrected iPSCs retained HBB expression as compared with the parental iPSCs line. Hence, this study offers another strategy in curing disease as corrected iPSCs display its normal functions and also can be used as a source of cells for transplantation in those patients having this disease (112).

In the light of several recent studies, it was revealed that for the correction of genetic diseases, CRISPR–cas9 can also be used in small mammal models. In rodents, desired mutation can be generated in single step by using the zygote injection of cas9 mRNA and sgRNA. For example, a study revealed that mice having mutated Crygc gene responsible for cataracts could be rescued by zygote injection of cas9, mRNA, and sgRNA (97).

Recently, a study revealed no off-target effects during gene editing in cynomolgus monkeys upon co-injection of cas9, mRNA, and sgRNA into one cell stage embryo (113). Hence, this achievement has considerably significant impact because monkeys are considered as model species for studying human diseases, and this strategy can help in developing new therapeutic approaches.

Moreover, some scientists, in 2016, reported that they develop new approach for gene editing (base editing), which irreversibly converts the one DNA base into another one without the requirement of a donor template or the cleavage of dsDNA. They engineered CRISPR–cas9 fusion with cytidine deaminase enzyme that has the ability to be programmed with a guide RNA, without the cleavage of dsDNA, and facilitates directly conversion of cytidine to uridine, thus effecting the substitution of C to T or (G to A). As a result of base editing, cytidine converts approximately within a window of five nucleotides, and corrects efficiently various point mutations relating to human disease (114).

Similar to cas9 gene-editing technique, a group of scientists took endonucleases from the Argonaute protein family which uses oligonucleotides for the degradation of invader’s genome. They reported that the *Natronobacterium gregoryi* Argonaute (NgAgo) is DNA-guided endonuclease, which play role in genome editing in human cells. NgAgo interact with ~24 nucleotides of 5′ phosphorylated single stranded guide DNA (gDNA), creating site specific dsDNA breaks. As in the case of cas9, PAM is not required by NgAgo–gDNA system. Upon preliminary characterization, it reveals that there is low tolerance to guide-target mismatches, whereas high efficiency observed in G + C rich genomic targets (115).

## CRISPR–Cas9 Potential to Target Diseases Developed by Epigenetic Alterations

Epigenetics is defined as: molecular mechanisms (heritable) that are involved in changing the gene expression without any alteration in DNA sequence (116). This mechanism consists of DNA methylation and histone modification that provides conducive environment for the stimulation of gene expression, which defines their cell proliferation as well as differentiation activity (117). The mammalian genome has CpG dinucleotide and DNA methylation usually occur at 5′ carbon of the cytosine ring within this dinucleotide (118). Three main DNA methyltransferases (DNMTs) are found in mammalian cells: DNMT1, DNMT3A, and DNMT3B. DNMT1 is involved in maintaining the pre-existing methylation patterns of DNA replication during cell divisions (119); however, DNMT3A and DNMT3B involved in the methylation of previously unmethylated CpGs yielding (120, 121). Another main type of epigenetic mechanism is histone modifications, which occur on histone protein-specific amino acid (122). To date, discovered histone modifications are acetylation, methylation, phosphorylation, ADP ribosylation, ubiquitylation, and SUMOylation. However, out of these, acetylation and methylation are the best known histone modification (123).

It has been established that epigenetic abnormalities are involved in the genesis as well as cancer cell progression. Among these abnormalities, common one is alteration in the methylation pattern of genomic DNA. Loss of DNA methylation globally along with hypermethylation at particular loci characterizes a major portion of human cancer. Overall, DNA hypomethylation could lead to the activation of genes that are silenced in normal cells and affect the stability of genome such as viral and parasitic transposons. However, DNA hypermethylation, which is gene-specific, is commonly found in the promoter regions and that results in abnormal silencing of PTEN, TP53, BRCA1, ATM, etc. (tumor suppressor genes) (124).

In the light of several studies, it was revealed that abnormalities in DNA methylation play an important role in the development of autoimmune diseases, such as systemic lupus erythematosus (SLE). It was reported that in active lupus and lupus like diseases, there is abnormal hypomethylation of whole T cell genomic DNA (125). When regulatory elements are hypomethylated then they may also be involved in abnormal perforin expression. Perforin is a cytotoxic molecule of CD4+ T cells from patients having active lupus. Perforin may contribute in spontaneous killing of macrophages or monocytes that characterizes lupus T cells (126).

These days, numerous non-coding RNA species such as micro RNA (miRNA), short interfering RNA (siRNA) are found. These RNA species involved in the activation or inhibition of genes that play role in the epigenetic regulation of various biological processes, such as growth and development (127). In various disease conditions, miRNAs are implicated and can be used as to target particular gene expression (up or down regulation) according to the requirement (128). Currently, successful editing of genetic switches is achieved by using CRISPR–cas9 system (129), and numerous miRNAs that play role in the progression and development of cancer can be targeted specifically with CRISPR–cas9 system (130). For example, DNTM1 and various other factors involved in epigenetic-silencing are excellent contestants to test such strategies. As compared with normal cells, cancerous cells are more prone to DNTM1 activity, so targeting this enzyme should result in limited side effects (131).

To treat cancerous and other deadly genetic diseases, CRISPR–cas9 system gives a strong hope due to its fabulous outcomes in genome-editing technology. The gene expression was suppressed when dCas9 (double mutant) bind with a repressor Krupple-associated box (KRAB); however, its specificity at genomic and heterochromatin level was not known until its binding reports with HS2 enhancer, having unique role in the expression of many globin genes. By observing the highly specific H3K9 trimethylation and limited chromatin accessibility of enhancer and promoter indicate that for the control of epigenome changes, each and every enhancer can be modified successfully (132).

## Future Dimensions

CRISPR–cas system has many useful applications regarding immunity and also its biological importance is not less exciting. For example, CRISPR–cas system can be acquired by phages in order to target the host defense systems; which are an evolutionary and puzzling turn of events (56). There are also some phages having genes that play role in the inhibition of CRISPR immunity (133). In *F. novicida*, cas9 can repress an endogenous lipoprotein gene in order to promote pathogenesis by preventing pro-inflammatory response of the host against this lipoprotein (45). CRISPR–cas systems also acts as a barriers against HGT (48, 57, 134), a fact that highlights significance of CRISPR immunity in the evolution of prokaryotes. Hence, one may wonder: now what is the next field for CRISPR (135)?

There is no doubt about role of cas9 in genome-editing technologies into medical applications, most importantly gene therapy which has the potential to make a significant impact on human health. Undoubtedly, the translation of cas9-based genome-editing technologies into medical applications, most notably gene therapy, has the potential to make a significant impact on human health (if off-target effects are somehow reduced to acceptable levels). The recent advancement in understanding the biochemical nature of cas9 targeting (136), along with structural insights into cas9:sgRNA complex formation (137, 138), help us in improving the molecular biological tools. In addition, if we look cursory on CRISPR-related publication and citation rate, it will show us that there is a globally awareness about the potential of CRISPR–cas systems, which indicate that this system will be used in many molecular biology laboratories, if not most. So, due to the acceleration of industrial exploitation, commercialization and financial investment(s) are setting the platform for a sustainable CRISPR revolution.

The cas9 system can be used in animal models for the correction of their genetic mutation, which, in turn, shows the significance of this system in the study of human diseases (113, 139). Studies relating to genetics help in identifying the several disease and trait-associating genetic variants, and it was found out that 93% variants are from outside the protein coding sequence. This suggests that aberrant regulation of gene expression along with non-coding RNAs is the key for causing diseases (140). Hence, techniques used for manipulating the gene expression could be important for disease research. For *in vivo* regulation of gene, CRISPR–cas9 system use as an alternative of RNAi for the study of gene function, modeling, and therapeutics. Noteworthy, CRISPR system have significant benefits when we deal with *in vivo* activation studies, such as multiple genes can be activated simply by expressing the several small sgRNAs.

For the delivery of CRISPR–cas9 system, currently DNA- and RNA-based injection technologies are used, like injection of such plasmids are available who has the ability of expressing cas9 and sgRNA. It is very essential to develop improved or alternative methods for the delivery of CRISPR–cas9 system into cell culture and organisms; so that this system can be used further for therapeutic purposes. For example, nanoparticles can be used for the delivery of viral vectors or nucleic acid which were previously used successfully for the treatment of genetic diseases of liver by using other genome-editing systems (141). Cas9 delivery on a plasmid or viral vector to a particular tissue in whole organisms is also a challenge and it must be resolved, if want to use cas9 for clinical purposes (47, 142–145).

In characterized model systems, if CRISPR–cas system is the representative of adaptive immunity then its overall impact on the host–virus population, genome trajectories, co-evolutionary dynamics and their role in evolution, and ecology of microbial population in nature is and will continue to be exciting. We also anticipate that there is further need to characterize CRISPR model systems, mechanism of action, their genetic elements, and functional modules.

## Conclusion

In conclusion, bacteria and archaea have evolved their adaptive immune system in order to regulate the exchange of invaders DNA. In adaptive immune system, CRISPR–cas systems have played an important role against foreign genetic elements. These systems also play key role in the survival and evolution of bacteria. Although, CRISPR–cas systems are diverse from each other in term of both phylogenetically and functionally, but still in order to cleave invaders DNA, these system relies on three common steps: new sequence or spacer integration, biogenesis of crRNA, and interference which is crRNA-guided. In addition to provide adaptive protection against invaders, self-targeting of CRISPR–cas systems can regulate the islands expulsion and genomic deletions that might play role in bacterial fitness. Also, self-targeting might play role in genome mosaicism, which ensures sufficient diversity within bacterial populations for rapid niche adaptation. Moreover, by using CRISPR-associated surveillance complexes, we can target any sequence of choice. Hence, these complexes open a new gateway or provide new opportunities about their use in various biotechnological and biomedical fields.

## Author Contributions

HH, MZS, HIH, ZI, SA, AS, MI, JL, and ZY help MABS in critical reading of this review article.

## Conflict of Interest Statement

The authors declare that the research was conducted in the absence of any commercial or financial relationships that could be construed as a potential conflict of interest. The reviewer CL and handling Editor declared their shared affiliation, and the handling Editor states that the process nevertheless met the standards of a fair and objective review.
